# Risk Signature Related to Immunotherapy Reaction of Hepatocellular Carcinoma Based on the Immune-Related Genes Associated With CD8^+^ T Cell Infiltration

**DOI:** 10.3389/fmolb.2021.602227

**Published:** 2021-03-19

**Authors:** Yiping Zou, Zhihong Chen, Hongwei Han, Shiye Ruan, Liang Jin, Yuanpeng Zhang, Zhengrong Chen, Zuyi Ma, Qi Lou, Ning Shi, Haosheng Jin

**Affiliations:** ^1^ Department of General Surgery, Guangdong Provincial People’s Hospital, Guangdong Academy of Medical Sciences, Guangzhou, China; ^2^ College of Medicine, Shantou University, Shantou, China; ^3^ The Second School of Clinical Medicine, Southern Medical University, Guangzhou, China

**Keywords:** CD8^+^ T cell, immune checkpoint inhibitors, prognosis, immune-related genes, hepatocellular carcinoma

## Abstract

**Background:** Hepatocellular carcinoma (HCC) is the most common histological type of liver cancer, with an unsatisfactory long-term survival rate. Despite immune checkpoint inhibitors for HCC have got glories in recent clinical trials, the relatively low response rate is still a thorny problem. Therefore, there is an urgent need to screen biomarkers of HCC to predict the prognosis and efficacy of immunotherapy.

**Methods:** Gene expression profiles of HCC were retrieved from TCGA, GEO, and ICGC databases while the immune-related genes (IRGs) were retrieved from the ImmPort database. CIBERSORT and WGCNA algorithms were combined to identify the gene module most related to CD8^+^ T cells in the GEO cohort. Subsequently, the genes in hub modules were subjected to univariate, LASSO, and multivariate Cox regression analyses in the TCGA cohort to develop a risk signature. Afterward, the accuracy of the risk signature was validated by the ICGC cohort, and its relationships with CD8^+^ T cell infiltration and PDL1 expression were explored.

**Results:** Nine IRGs were finally incorporated into a risk signature. Patients in the high-risk group had a poorer prognosis than those in the low-risk group. Confirmed by TCGA and ICGC cohorts, the risk signature possessed a relatively high accuracy. Additionally, the risk signature was demonstrated as an independent prognostic factor and closely related to the CD8^+^ T cell infiltration and PDL1 expression.

**Conclusion:** A risk signature was constructed to predict the prognosis of HCC patients and detect patients who may have a higher positive response rate to immune checkpoint inhibitors.

## Introduction

Being one of the most aggressive malignant tumors in the world, hepatocellular carcinoma (HCC) is the fourth leading cause of cancer-related mortality, causing almost 800,000 deaths annually (Bray et al., 2018). HCC is commonly developed on the background of chronic liver diseases, such as chronic hepatitis B virus (HBV) and hepatitis C virus (HCV) infection, chronic alcohol consumption, and metabolic disorders. The main treatment strategy for HCC is surgical resection. However, due to the hidden symptoms in the early stage, a high percentage of patients with HCC are diagnosed in the advanced stage and unable to undergo surgery (Forner et al., 2018). Nowadays, there exist only two oral multikinase inhibitors, Sorafenib and lenvatinib (Cheng et al., 2009; Kudo et al., 2018), which are recommended by the National Comprehensive *Cancer* Network (NCCN) guidelines as the first-line therapy for patients with advanced HCC (Hepatobiliary Cancers, 2019). Nevertheless, only a few patients who take the drugs can achieve an effective response rate. Therefore, the exploration of novel potential markers has become an important focus of HCC research recently.

Over the past decade, immunotherapies based on the principle of the immune checkpoint blockade have developed rapidly and achieved impressive success in several types of cancers, such as melanoma, lung cancer, and liver cancer (Havel et al., 2019). In September 2017, nivolumab, a programmed cell death protein 1 (PD-1) antibody was firstly approved for the second-line treatment in patients with advanced HCC by the Food and Drug Administration (FDA) (El-Khoueiry et al., 2017). Subsequently, other PD-1 inhibitors (pembrolizumab and camrelizumab) have got favorable results in the treatment of advanced HCC in recent clinical trials (Zhu et al., 2018; Qin et al., 2020). Recently, systemic therapy that combine atezolizumab (anti-PD-L1) with bevacizumab (anti-VEGF) resulted in better overall and progression-free survival than sorafenib and included in the newly first-line treatment for advanced liver cancer (Finn et al., 2020). However, nearly 80% of HCC patients still have not ideal response to PD-1 inhibitors (Macek Jilkova et al., 2019). Thus, in order to improve the efficacy of immunotherapy, it is highly necessary to identify predictive factors of good response to PD-1 inhibitors in HCC patients.

High infiltration level of CD8+T cells has been regarded as a marker of favorable prognosis in most solid tumors, including HCC (Mahmoud et al., 2011; Erdag et al., 2012; Xu et al., 2019; Orhan et al., 2020). Furthermore, high infiltration level of CD8+T cells could also improve the responses to chemotherapy and immunotherapy in solid tumors (Danilova et al., 2016; Riaz et al., 2017). Therefore, the biomarkers related to CD8^+^ T cell infiltration could be potentially conducive to predict the prognosis and response to immunotherapy in HCC. In the present study, we identified a hub module of immune-related genes (IRGs) related to CD8^+^ T cell infiltration level in HCC, and a risk signature based on genes of the hub module was constructed and validated by bioinformatics analysis. The risk signature was related to the prognosis of HCC patients and had the potential function to predict the efficacy of immunotherapy.

## Materials and Methods

### Data Acquisition

Three HCC data sets from public databases were obtained and analyzed in the present study, in which one microarray data set (GSE63898) containing 288 HCC samples from the Gene Expression Omnibus (GEO), and the other two transcriptome sequencing data sets were downloaded from The *Cancer* Genome Atlas (TCGA) data portal (https://cancergenome.nih.gov/) and International *Cancer* Genome Consortium (ICGC) data portal (https://icgc.org/). The TCGA-LIHC (liver hepatocellular carcinoma) cohort included 370 HCC samples while the ICGC-LIRI-JP (liver cancer, RIKEN, Japan) cohort comprised 243 HCC samples. A total of 2,498 IRGs were obtained from The Immunology database and Analysis Portal (ImmPort) (https://immport.niaid.nih.gov) (Bhattacharya et al., 2014).

### Estimation of Tumor-Infiltrating Immune Cells in GSE63898 and TCGA

The CIBERSORT algorithm, a useful tool to estimate the abundances of special cells in mixed tissues, was applied to calculate the fractions of the 22 types of tumor-infiltrating immune cells (TIICs) based on gene expression data via convolution algorithm (Newman et al., 2015). The estimated results of the infiltration of the immune cells with *p*-value less than 0.05 were considered as the more reliable estimation by CIBERSORT and were retained for further analysis.

### Construction of Co-expression Network and Module Correlated Analysis

HCC samples from GSE63898 were screened according to the estimation effect of CIBERSORT results and their gene expression data of 2,498 IRGs were used to construct a weighted co-expressed network analysis (WGCNA) (Langfelder & Horvath, 2008). The correlation between gene modules and CD8^+^ T cells infiltration was evaluated by Pearson correlation coefficients in WGCNA. The hub module was defined as the module with the highest correlation coefficient. To further clarify the molecular mechanism and functions underlying the genes of the hub module, Gene Ontology (GO) term analysis and Kyoto Encyclopedia of Genes and Genomes (KEGG) pathway analysis were performed.

### Construction and Evaluation of Risk Signature Based on Hub Module

The univariate, least absolute shrinkage and selection operator (LASSO), and stepwise multivariate Cox regression analyses were applied in turn to identify the genes significantly related to overall survival (OS) in TCGA-LIHC and construct a risk signature (Tibshirani, 1997). The individual risk score of each HCC patient could be calculated using the following formula: risk score = Ʃ(βi × Expi), in which i was referred to the number of prognostic genes, *β* was the regression coefficient value for each gene and Exp meant gene expression level, respectively. After excluding samples without defined survival data, we divided the HCC samples into high-risk or low-risk groups according to the cutoff value decided by the median risk score. Subsequently, Kaplan–Meier (KM) survival curves were plotted and time-dependent receiver operational feature curve (ROC) analyses were conducted to evaluate the predictive accuracy of the novel model. In addition, the risk signature was verified by the external cohort from ICGC-LIRI-JP.

### Identification of the Relationship Between Risk Signature and Immunotherapy Related Biomarkers

CD8^+^ T cells and PD-L1 expression were identified as two potential immunotherapy related biomarkers, which had been confirmed by the past researches (Havel et al., 2019). To further investigate the relationship of a specific gene and CD8^+^ T cells infiltration, we conducted TIMER (https://cistrome.shinyapps.io/timer/) database analysis, which is an online database aimed to analyze the immune infiltration in different 32 cancer types from the TCGA database (Li et al., 2017). The correlations between genes from risk signature and immunotherapy related markers were evaluated by the Spearman’s correlation analysis. The statistical significance of differences in immunotherapy related markers between samples in high-risk and low-risk groups were analyzed by the Wilcoxon signed-rank test and the boxplots were constructed to visualize the results.

### Statistical Analysis

All statistical analyses were conducted using R version 4.0.0 software (http://www.r-project.org/). A *p*-value of <0.05 was considered statistically significant.

## Results

### Identification of Hub Module Associated With CD8^**+**^ T Cell Infiltration in HCC

**FIGURE 1:**
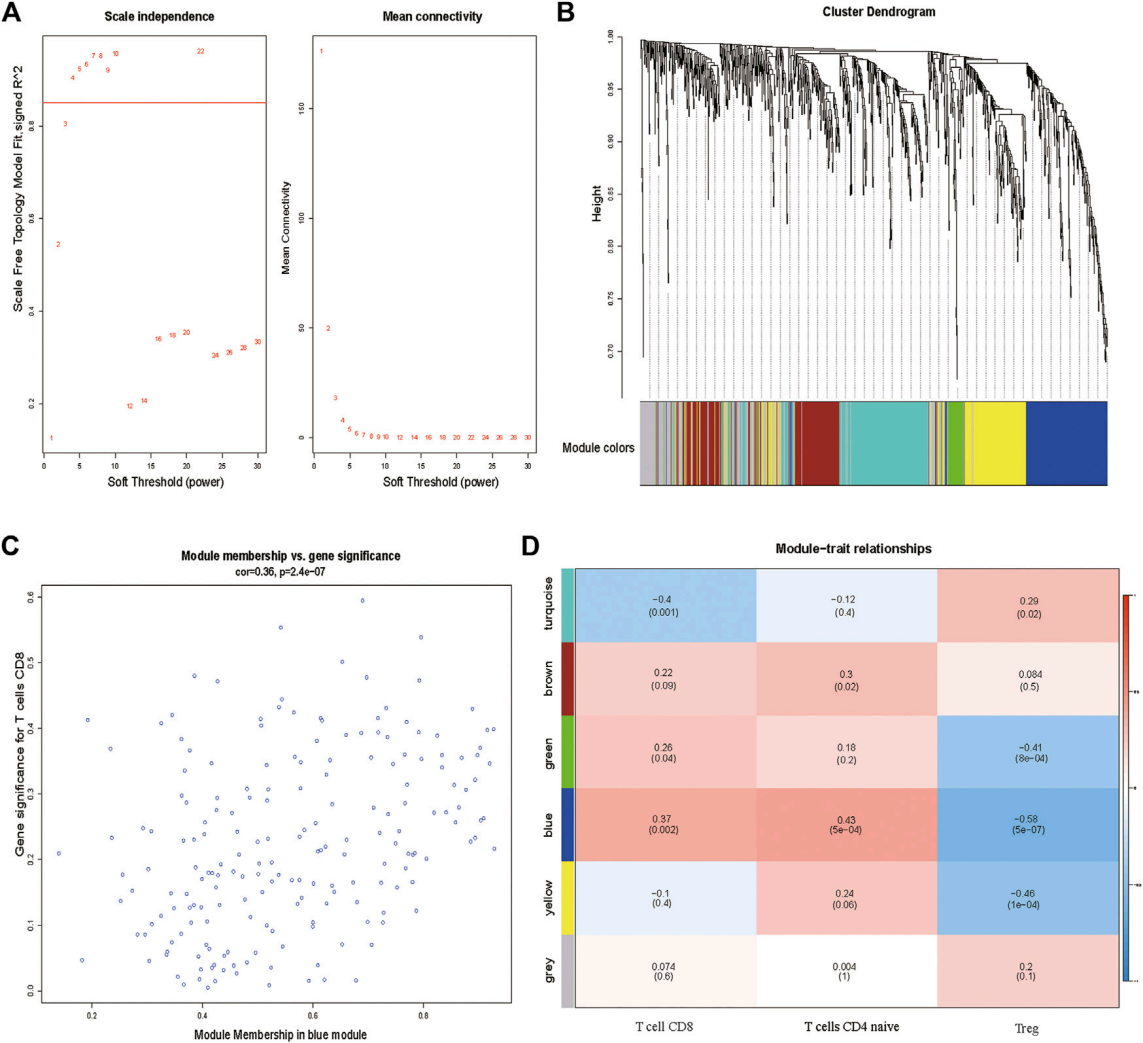
**(A)** Determination of soft-thresholding power in the WGCNA. **(B)** Clustering dendrogram of 2,498 IRGs and six merged modules from 288 HCC samples. **(C)** Blue module MM and GS relationship scatter plot. **(D)** Heatmap of the correlation between module eigengenes and clinical traits. The upper row in each cell indicates the correlation coefficient ranging from −1 to 1 of the correlation between a certain module and clinical trait. IRGs, immune-related genes. MM, module membership. GS, gene significance.

Using the R package “CIBERSORT”, the fractions of 22 TIICs in HCC samples from the GSE63898 were calculated. Then, the fraction of subtype of CD8^+^ T cells in every sample were selected as the trait data of WGCNA. Additionally, the expression values of the 2,498 IRGs were employed to construct a gene co-expression network using the R package “WGCNA”. The weighted method meant the power operation of the correlation value among gene expression, which could make the correlation value of two genes with the similar expression pattern stronger and make the correlation value between two genes with the unsimilar expression pattern weaker. The appropriate range of power was decided by the soft threshold, *β*. A value of *β* = 4 was identified as a soft threshold to establish a gene regulatory network (Figure 1A). After constructing a hierarchical clustering tree using a dynamic hybrid cutting method, six modules (turquoize, brown, green, blue, yellow, and gray) were generated (Figure 1B). Each leaf on the tree represents a gene, and genes with similar expression pattern were gathered to form a branch of the tree, represented a gene module. Among the six modules, the blue module containing 195 genes was highly correlated to CD8^+^ T cells infiltration (r = 0.37, *p* = 0.002) and was selected as the hub module (Figures 1C,D).

**FIGURE 2:**
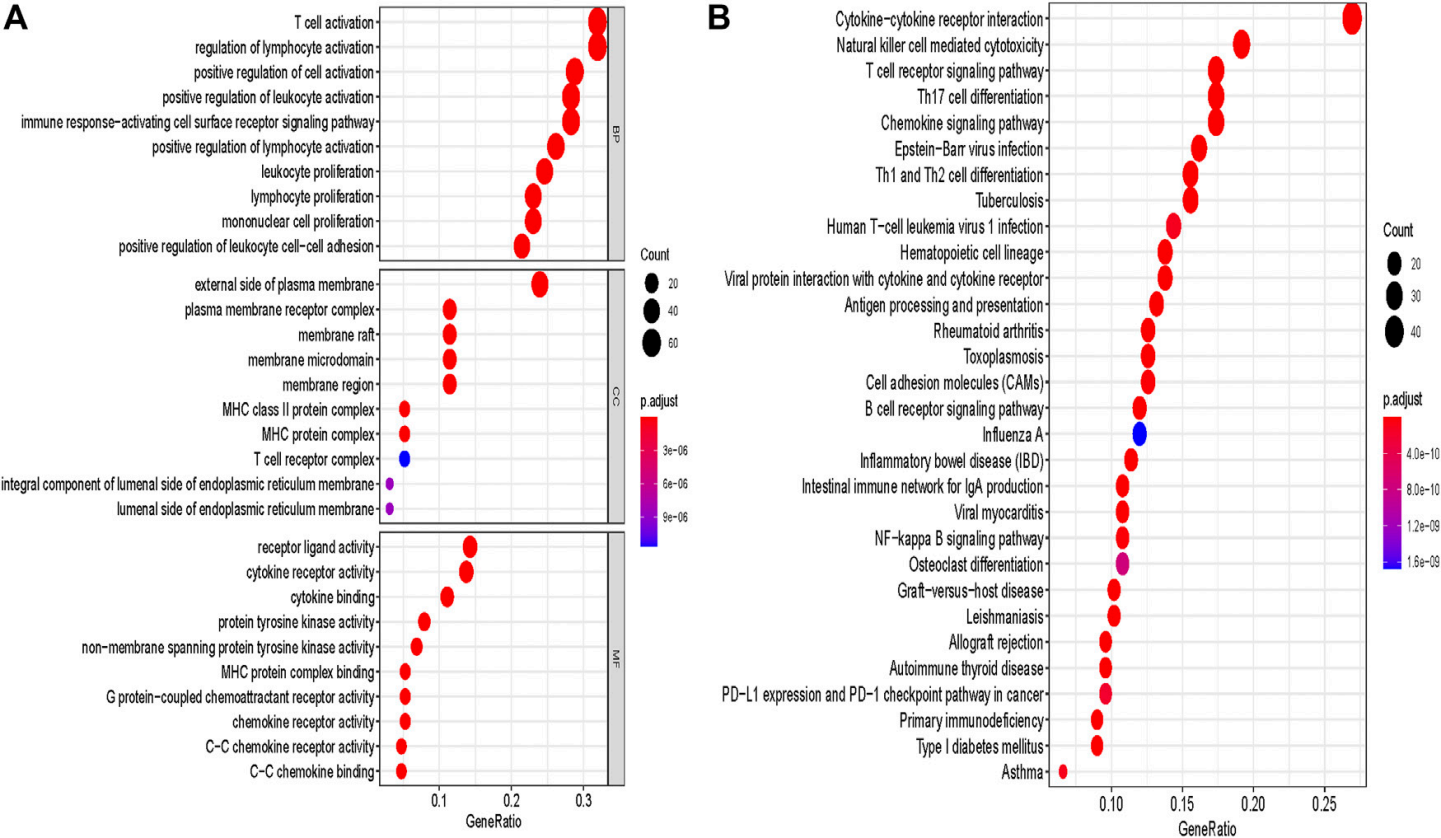
GO and pathway enrichment analysis of blue module genes. **(A)** GO analysis. **(B)** KEGG pathway analysis. GO, Gene Ontology. KEGG, Kyoto Encyclopedia of Genes and Genomes.

Furthermore, in order to analyze the biological function of the hub module, genes of the blue module were extracted for GO term and KEGG pathway annotation analyses. Notably, the most enriched terms of GO and KEGG analysis were immune-related terms, especially containing T cell-related terms including “T cell activation”, “T cell receptor signaling pathway” and “PD−L1 expression and PD−1 checkpoint pathway in cancer” (Figures 2A,B). This result further confirmed that genes of the blue module were highly associated with activation and function of T cells.

### Construction of Prognostic Signature Based on Genes of the Blue Module From TCGA

**FIGURE 3:**
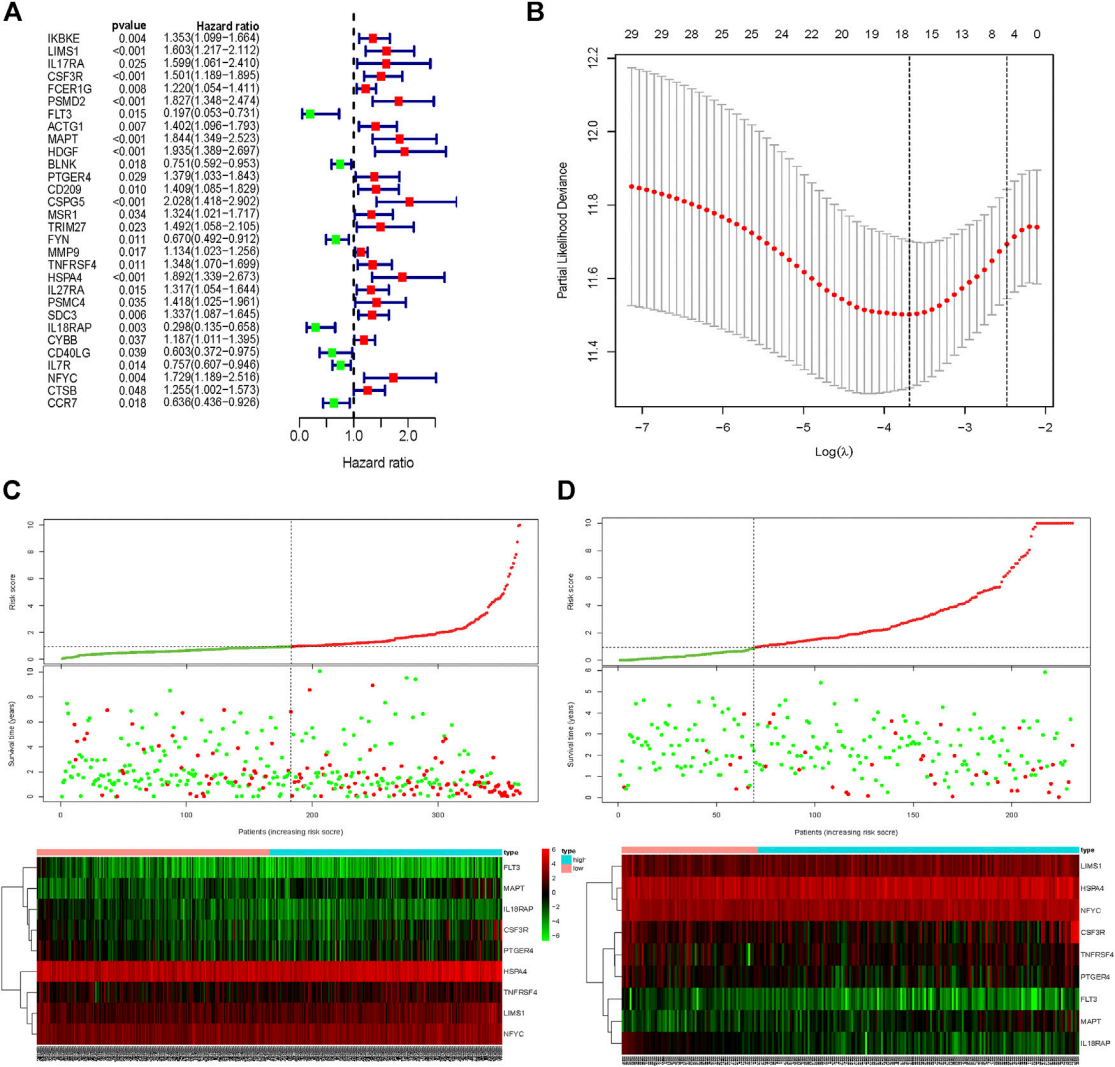
Signature-based risk score is a promising marker in the training and validation cohorts. **(A)** Univariate COX regression for OS of IRGs in TCGA cohort. **(B)** LASSO COX regression for OS of IRGs in the TCGA cohort. Risk score distribution, survival overview, and heatmap for patients in the TCGA **(C)** and ICGC **(D)** cohorts assigned to high- and low-risk groups based on the risk score. OS, Overall survival. LASSO, least absolute shrinkage and selection operator.

The univariate Cox regression analysis was used to identify the genes in the blue module related to the OS of HCC patients from TCGA-LIHC (Figure 3A). A total of 30 genes were identified and further analyzed by the LOSSO COX regression model to discriminate stable markers and avoid overfitting the model (Figure 3B). Subsequently, 17 genes detected via the LOSSO COX regression were evaluated by stepwise multivariate Cox regression analysis. Finally, we got nine prognosis genes which were used to build a risk signature. The risk score of each patient could be calculated as follows formula: risk score = (0.06868 × expression value of LIMS1) + (0.09839 × expression value of CSF3R) + (-1.3727 × expression value of FLT3) + (0.2712×expression value of MAPT) + (0.09629×expression value of TNFRSF4) + (0.02645 × expression value of HSPA4) + (-1.7299 × expression value of IL18RAP) + (0.04982 × expression value of NFYC) + (0.08851 × expression value of PTGER4) (Table 1).

**TABLE 1:** Multivariate COX for OS of nine IRGs in HCC.

Gene	Coef	HR	HR.95% L	HR.95% H	*p* value
LIMS1	0.068684	1.071097	1.012443	1.13315	0.016833
CSF3R	0.098386	1.103,389	1.038254	1.17261	0.001529
FLT3	−1.37271	0.25342	0.039602	1.621,655	0.147,204
MAPT	0.271,202	1.311,539	1.067857	1.610,829	0.00971
TNFRSF4	0.096287	1.101,075	1.000201	1.212,123	0.049523
HSPA4	0.026452	1.026805	1.007806	1.046162	0.005503
IL18RAP	−1.72992	0.177,299	0.059277	0.530,306	0.00197
NFYC	0.04982	1.051082	0.984,617	1.122,034	0.134,961
PTGER4	0.088509	1.092544	0.984,496	1.212,451	0.095741

Coef: co-efficient, HR: hazard ratio.

HCC patients from TCGA were assigned to high-risk and low-risk groups according to the cutoff value of the median risk score. Comparing the survival status between two risk groups, we found that the prognosis of the high-risk group was poorer than the low-risk group (Figure 3C). More death occurred in the high-risk group than the low-risk group. In addition, the risk signature was validated externally by an independent dataset from ICGC. By using the same formula in the TCGA dataset, the risk score of each patient in 231 HCC patients with follow-up from ICGC was calculated. By taking the same cutoff value, the HCC patients in ICGC were also divided into high-risk (n = 162) and low-risk (n = 69) groups. A similar result was observed between the TCGA and ICGC cohorts, that the high-risk group had a poorer prognosis than the low-risk group in the ICGC cohort (Figure 3D).

### The Prognostic Performance of Risk Signature

**FIGURE 4:**
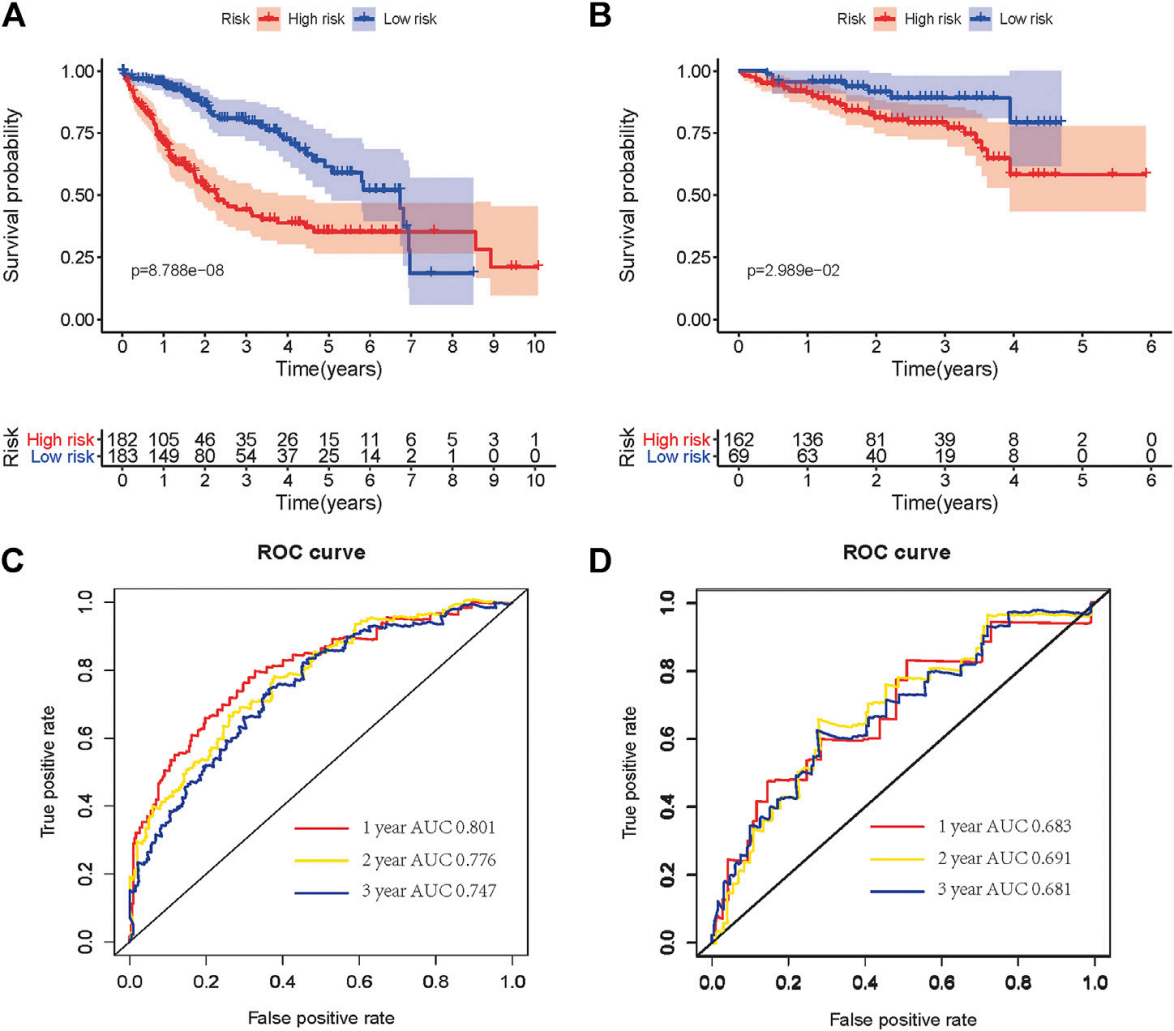
Kaplan–Meier OS curves for patients in the TCGA **(A)** and ICGC **(B)** cohorts assigned to high- and low-risk groups based on the risk score. ROC curves showed the predictive efficiency of the risk signature for patients in the TCGA **(C)** and ICGC **(D)** cohorts on OS. OS, overall survival. ROC, receiver operational feature curve.

The Kaplan–Meier survival curve was used to show a comparison of the OS between the high-risk and low-risk groups in HCC patients from TCGA and ICGC cohorts (Figures 4A,B). A significant difference in OS between high-risk and low-risk groups was found in the TCGA cohort (*p* = 8.788e-08) and ICGC cohort (*p* = 0.02989), respectively. Additionally, the area under the ROC curve (AUC) of the time-dependent ROC curve was plotted to evaluate the prognostic ability of the risk signature, and a higher AUC indicates a more accurate model. In the TCGA cohort, the AUCs of the risk signature corresponding to 1, 2, and 3 years of survival were 0.801, 0.776, and 0.747 (Figure 4C). In the ICGC cohort, the AUCs of the risk signature corresponding to 1, 2, and 3 years of survival were 0.683, 0.691, and 0.681 (Figure 4D). These results demonstrated that the risk signature had relatively high sensitivity and specificity in both the TCGA and external validation ICGC cohorts, which indicated the risk signature could be used to predict OS in patients with HCC reliably.

### The Risk Signature Is an Independent Prognostic Factor for Patients With HCC

The univariate and multivariate Cox regression analyses were further used to assess prognostic significances of risk signature and several clinicopathologic characteristics in the TCGA cohort. As described in Table 2, the result of univariate Cox regression analysis showed that AJCC TNM stage (HR: 1.672, 95%CI: 1.359–2.056, *p* < 0.001), T stage (HR: 1.652, 95%CI: 1.357–2.011, *p* < 0.001), and IAGs risk score (HR: 1.159, 95%CI: 1.116–1.203, *p* < 0.001) were correlated with the prognosis of HCC patients. Besides, the result of multivariate Cox regression analysis indicated that only IAGs risk score (HR: 1.171, 95%CI: 1.123–1.222, *p* < 0.001) was independently associated with the prognosis of HCC patients. Therefore, the IRGs risk signature could be considered as an independent prognostic factor for patients with HCC.

**TABLE 2:** Cox regression of prognostic variables for the OS in the TCGA cohort.

	Univariate analysis		Multivariate analysis	
Variable	HR (95%CI)	*p*-value	HR (95% CI)	*p*-value
Age	1.010 (0.995–1.025)	0.177	1.011 (0.996–1.027)	0.155
Gender	0.820 (0.557–1.209)	0.317	1.029 (0.685–1.546)	0.981
Grade	1.121 (0.868–1.446)	0.382	1.104 (0.841–1.450)	0.477
Stage	1.672 (1.359–2.056)	<0.001	1.177 (0.505–2.739)	0.706
T Stage	1.652 (1.357–2.011)	<0.001	1.467 (0.656–3.279)	0.351
Risk score	1.159 (1.116–1.203)	<0.001	1.171 (1.123–1.222)	<0.001

HR, hazard ratio; CI, confidence interval.

### The Close Relationship Between the Risk Signature and Immunotherapy Related Biomarkers in HCC

**FIGURE 5:**
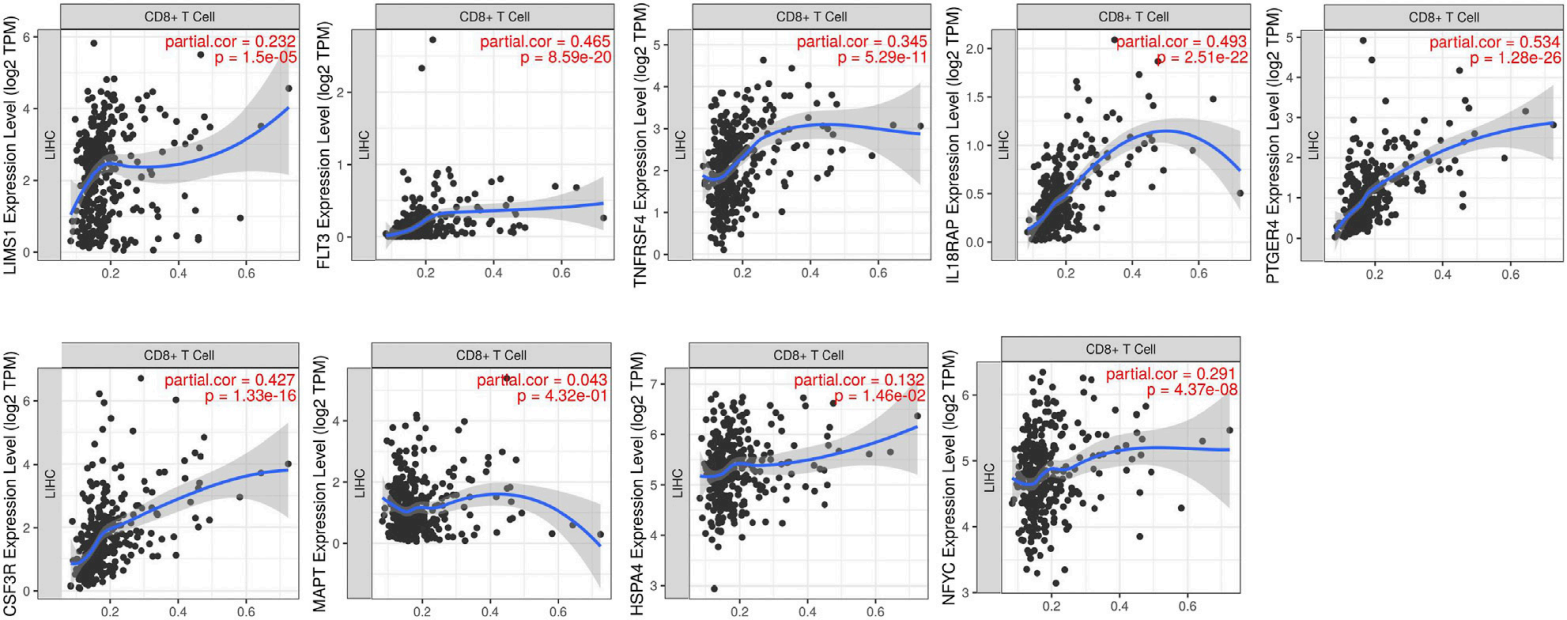
Relationship between expressions of nine IRGs and CD8^+^ T cells infiltration. IRGs, immune-related genes.

**FIGURE 6:**
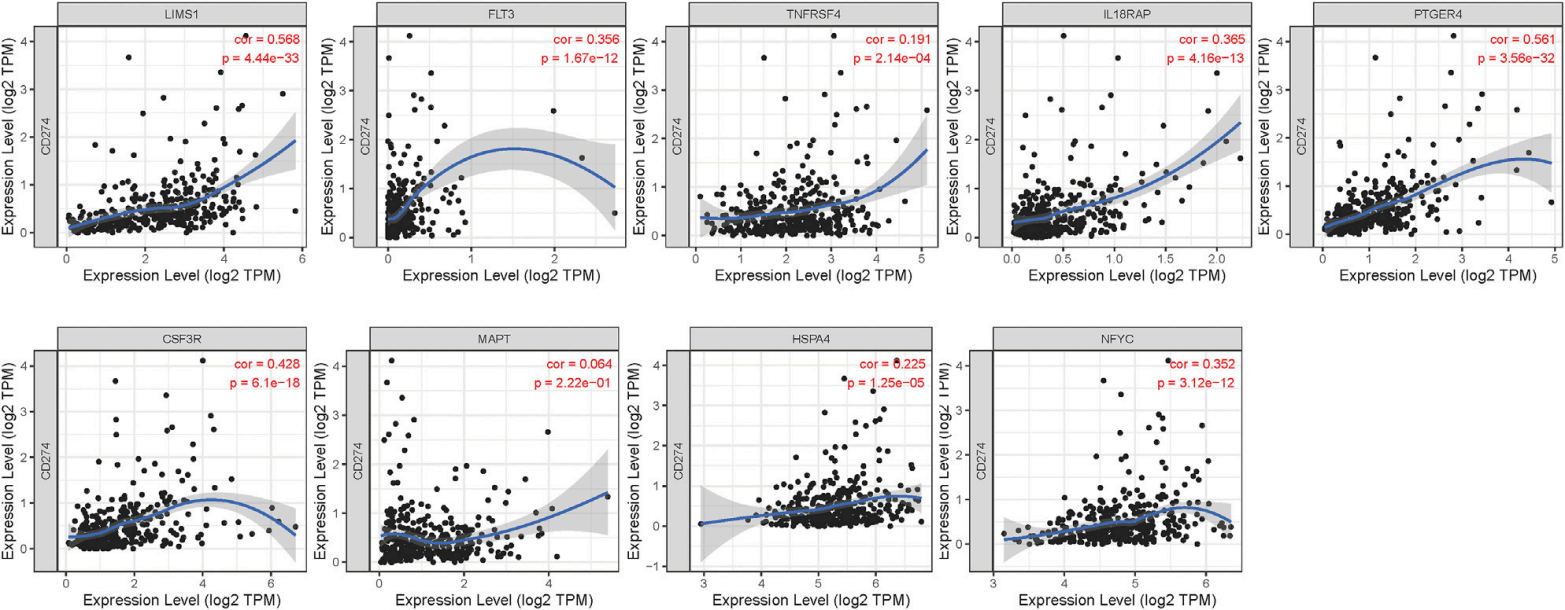
Relationship between expressions of nine IRGs and expression of PDL1 (CD274). IRGs, immune-related genes.

We explored the relationships between the expressions of genes in risk signature and immunotherapy related biomarkers in HCC using the TIMER database. Except of MAPT (r = 0.043, *p* = 4.32e-01), the expression level of LIMS1 (r = 0.232, *p* = 1.5e-05), FLT3 (r = 0.465, *p* = 8.59e-20), TNFRSF4 (r = 0.345, *p* = 5.29e-11), IL18RAP (r = 0.493, *p* = 2.51e-22), PTGER4 (r = 0.534, *p* = 1.28e-26), CSF3R (r = 0.427, *p* = 1.33e-16), HSPA4 (r = 0.132, *p* = 1.46e-02), NFYC (r = 0.291, *p* = 4.37e-08) were positively correlated with immune cells infiltration levels of CD8^+^ T cell (Figure 5). Similarly, the expression level of LIMS1 (r = 0.568, *p* = 4.44e-33), FLT3 (r = 0.356, *p* = 1.67e-12), TNFRSF4 (r = 0.191, *p* = 2.14e-04), IL18RAP (r = 0.365, *p* = 4.16e-13), PTGER4 (r = 0.561, *p* = 3.56e-32), CSF3R (r = 0.428, *p* = 6.1e-18), HSPA4 (r = 0.225, *p* = 1.25e-05), NFYC (r = 0.352, *p* = 3.12e-12) were positively correlated with PDL1 expression in HCC, except of MAPT (r = 0.064, *p* = 2.22e-01) (Figure 6).

**FIGURE 7:**
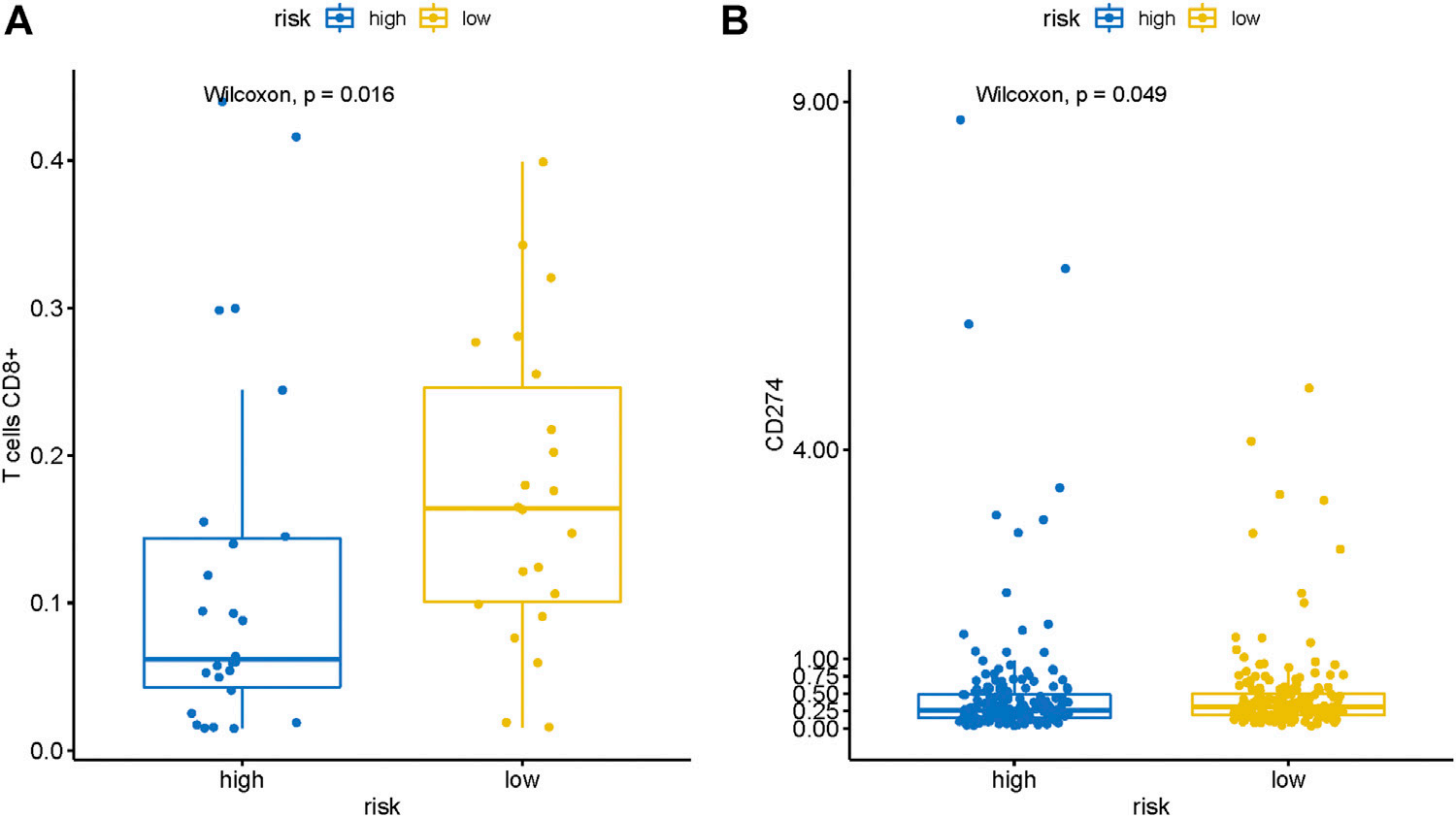
The risk signature is closely related to CD8^+^ T cells infiltration and PDL1 expression of HCC. Wilcoxon signed-rank test showed that the **(A)** CD8^+^ T cells infiltration and **(B)** PDL1 expression in the patients with the low-risk group were notably higher than that in patients with the high-risk group.

Then we employed CIBERSORT to further estimate the TIICs proportions of HCC in the TCGA cohort. Then we explored the difference of infiltration level of CD8^+^ T cells and expression of PDL1 (CD274) between the high-risk and low-risk groups. The results of the Wilcoxon test showed that the infiltration level of CD8^+^ T cells (*p* = 0.016) and expression of PDL1 (*p* = 0.048) were significantly higher in the low-risk group than the high-risk group (Figures 7A,B). Previous researches had revealed that a higher positive immunotherapy response rate was found in malignant tumors with higher infiltration levels of CD8+T cells and higher expression of PDL1 (Herbst et al., 2014; Riaz et al., 2017). Consequently, we could suppose that HCC patients in the low-risk group might have a higher reaction rate to immune checkpoint inhibitors, which indicated that the risk signature may also possess the potential ability to predict the efficacy of immunotherapy.

## Discussion

Hepatocellular carcinoma is the most common histological type of liver cancer, with an unsatisfactory long-term survival rate (Forner et al., 2018; Kulik & El-Serag, 2019). Immune checkpoint inhibitors have achieved success in clinical trials of advanced HCC (El-Khoueiry et al., 2017; Zhu et al., 2018; Qin et al., 2020). However, the low positive reaction rate is still one of the main obstacles to the promotion and application of immunotherapy. Previous studies found that CD8^+^ T cell played a critical role in immunotherapy and was correlated to prognosis in HCC (Mahmoud et al., 2011; Erdag et al., 2012; Danilova et al., 2016; Riaz et al., 2017; Xu et al., 2019; Orhan et al., 2020). Therefore, we sought to identify genes associated with CD8^+^ T cells in HCC and related to prognosis and immunotherapy efficacy prediction.

In the present study, the gene expression matrix of IRGs from GSE63898 was applied to construct the co-expression network and calculate the infiltration level of TIICs in HCC. Then the gene module most related to CD8^+^ T cells was identified by the WGCNA algorithm. Enrichment analysis of the GO and KEGG terms indicated that the hub module was highly related to immunologic mechanism and function, especially the pathways relevant to T cell and immune checkpoints in cancer. Moreover, univariate, LASSO, and multivariate Cox analyses were used to filter the prognostic genes and establish a risk signature for predicting the prognosis of HCC patients. The external validation of the ICGC-RIKEN-JP cohort proved that the risk signature had good accuracy in the prediction of the survival prognosis of HCC patients. Finally, significantly higher levels of CD8^+^ T cell infiltration and PDL1 expression were signfound in the low-risk group, suggesting a higher positive reaction rate to immune checkpoint inhibitors may be displayed in the low-risk group.

There were nine IRGs corrected with CD8^+^ T cells identified to construct the risk signature. These genes have been studied in the field of cancer. LIMS1 was originally confirmed as a marker for senescent erythrocytes and widely expressed in mammalian cells (Rearden, 1994). A recent study had found that LSMS1 could promote the survival of pancreatic cancer cells under oxygen-glucose deprivation condition by enhancing HIF1A protein translation and activating AKT/TOR signaling (Huang et al., 2019). CSF3R is a driver of neutrophil differentiation, proliferation, and activation following the combination with granulocyte colony-stimulating factor, resulting in the activation downstream signaling of tyrosine kinases (Nicholson et al., 1994). FLT3, a class III receptor tyrosine kinase, drives cellular proliferation through activation of the MAPK, PI3K, and STAT5 signaling pathways (Takahashi, 2011). MAPT, mainly expressed in neuronal cells, lymphocytes, and epithelial cells, is associated with survival in prostate cancer and promotes bicalutamide resistance (Sekino et al., 2020). Multiple studies have demonstrated that TNFRSF4 can work as a therapeutic agent and play a significant role in immunotherapy of preclinical tumor models (Linch et al., 2015; Aspeslagh et al., 2016). Recent research reported that HSPA4 is a tumor membrane antigen that can promote tumor metastasis by activating the NF-κB pathway in tumor cells once ligated with the pathogenic anti-HSPA4 IgG (Gu et al., 2019). Lin, et al. indicated that the knockdown of IL18RAP inhibited cell proliferation by causing cell cycle arrest in extranodal natural killer T-cell lymphoma cells, which might be a potential therapeutic target (Lin et al., 2020). NFYC was previously identified as an important regulator in tumorigenesis of choroid plexus carcinoma (Tong et al., 2015). Heinrichs, et al. revealed that the upregulated expression of PTGER4 in gastric tissue is related to the initiation of gastric cancer (Heinrichs et al., 2018).

In recent years, the identification of prognostic gene signature for HCC has been noted in many researches (Chen et al., 2020; Hu et al., 2020; Zhu et al., 2020). However, to the best of our knowledge, none of them constructed a risk signature based on IRGs related to CD8^+^ T cells. Compared with these models, our model not only has a good prediction accuracy in survival prognosis. but also possesses the potential ability to predict the efficacy of immunotherapy. Nevertheless, this hypothesis still needs to be further verified by randomized controlled trials of immunotherapy. In addition, the interaction among these nine genes and their potential role in predicting the response to immunotherapy for HCC still need to be further investigated.

In conclusion, our study identified risk signature based on nine IRGs to predict the prognosis of HCC and distinguish the patients who might have a better response to immunotherapy. Specifically, the prognosis of HCC patients in the low-risk group is relatively satisfactory, and they might have a higher response rate to immune checkpoint inhibitors.

## Data Availability

Publicly available datasets were analyzed in this study. This data can be found in The *Cancer* Genome Atlas (TCGA) database (https://portal.gdc.cancer.gov/), Gene Expression Omnibus (GEO) database (https://www.ncbi.nlm.nih.gov/geo/) and International *Cancer* Genome Consortium (ICGC) data portal (https://icgc.org/).

## References

[B1] Aspeslagh S. Postel-Vinay S. Rusakiewicz S. Soria J. C. Zitvogel L. Marabelle A. (2016). Rationale for anti-OX40 cancer immunotherapy. Eur. J. Cancer 52, 50–66. 10.1016/j.ejca.2015.08.021 26645943

[B2] Bhattacharya S. Andorf S. Gomes L. Dunn P. Schaefer H. Pontius J. (2014). ImmPort: disseminating data to the public for the future of immunology. Immunol. Res. 58, 234–239. 10.1007/s12026-014-8516-1 24791905

[B3] Bray F. Ferlay J. Soerjomtaram I. Siegel R. Torre L. Jemal A. (2018). Global cancer statistics 2018: GLOBOCAN estimates of incidence and mortality worldwide for 36 cancers in 185 countries. CA Cancer J. Clin. 68, 394–424. 10.3322/caac.21492 30207593

[B4] Chen W. Ou M. Tang D. Dai Y. Du W. Shi R. (2020). Identification and validation of immune-related gene prognostic signature for hepatocellular carcinoma. J. Immunol. Res., 5494858. 10.1155/2020/5494858 32211443 PMC7081044

[B5] Cheng A. L. Kang Y. K. Chen Z. Tsao C. J. Qin S. Kim J. S. (2009). Efficacy and safety of sorafenib in patients in the Asia-Pacific region with advanced hepatocellular carcinoma: a phase III randomised, double-blind, placebo-controlled trial. Lancet Oncol. 10, 25–34. 10.1016/S1470-2045(08)70285-7 19095497

[B6] Danilova L. Wang H. Sunshine J. Kaunitz G. J. Cottrell T. R. Xu H. (2016). Association of PD-1/PD-L axis expression with cytolytic activity, mutational load, and prognosis in melanoma and other solid tumors. Proc. Natl. Acad. Sci. U.S.A. 113, E7769–E77. 10.1073/pnas.1607836113 27837027 PMC5137776

[B7] El-Khoueiry A. B. Sangro B. Yau T. Crocenzi T. S. Kudo M. Hsu C. (2017). Nivolumab in patients with advanced hepatocellular carcinoma (CheckMate 040): an open-label, non-comparative, phase 1/2 dose escalation and expansion trial. Lancet 389, 2492–2502. 10.1016/S0140-6736(17)31046-2 28434648 PMC7539326

[B8] Erdag G. Schaefer J. T. Smolkin M. E. Deacon D. H. Shea S. M. Dengel L. T. (2012). Immunotype and immunohistologic characteristics of tumor-infiltrating immune cells are associated with clinical outcome in metastatic melanoma. Cancer Res. 72, 1070–1080. 10.1158/0008-5472 22266112 PMC3306813

[B9] Finn R. S. Qin S. Ikeda M. Galle P. R. Ducreux M. Kim T. Y. (2020). Atezolizumab plus Bevacizumab in unresectable hepatocellular carcinoma. N. Engl. J. Med. 14, 1894–1905. 10.1056/NEJMoa1915745 32402160

[B10] Forner A. Reig M. Bruix J. (2018). Hepatocellular carcinoma. Lancet 391, 1245–1255. 10.1016/S0140-6736(18)30010-2 29307467

[B11] Gu Y. Liu Y. Fu L. Zhai L. Zhu J. Han Y. (2019). Tumor-educated B cells selectively promote breast cancer lymph node metastasis by HSPA4-targeting IgG. Nat. Med. 25, 312–322. 10.1038/s41591-018-0309-y 30643287

[B12] Havel J. J. Chowell D. Chan T. A. (2019). The evolving landscape of biomarkers for checkpoint inhibitor immunotherapy. Nat. Rev. Cancer 19, 133–150. 10.1038/s41568-019-0116-x 30755690 PMC6705396

[B13] Heinrichs S. K. M. Hess T. Becker J. Hamann L. Vashist Y. K. Butterbach K. (2018). Evidence for PTGER4, PSCA, and MBOAT7 as risk genes for gastric cancer on the genome and transcriptome level. Cancer Med. 7, 5057–5065. 10.1002/cam4.1719 30191681 PMC6198243

[B14] Hepatobiliary Cancers (2019). NCCN clinical practice guidelines in oncology (NCCN guidelines). Version 2. (Accessed March 6 2019).

[B15] Herbst R. S. Soria J. C. Kowanetz M. Fine G. D. Hamid O. Gordon M. S. (2014). Predictive correlates of response to the anti-PD-L1 antibody MPDL3280A in cancer patients. Nature 515, 563–567. 10.1038/nature14011 25428504 PMC4836193

[B16] Hu B. Yang X. B. Sang X. T. (2020). Development of an immune-related prognostic index associated with hepatocellular carcinoma. Aging 12, 5010–5030. 10.18632/aging.102926 32191631 PMC7138589

[B17] Huang C. Li Y. Li Z. Xu Y. Li N. Ge Y. (2019). LIMS1 promotes pancreatic cancer cell survival under oxygen-glucose deprivation conditions by enhancing HIF1A protein translation. Clin. Cancer Res. 25, 4091–4103. 10.1158/1078-0432.CCR-18-3533 30679163 PMC6759834

[B18] Kudo M. Finn R. S. Qin S. Han K. H. Ikeda K. Piscaglia F. (2018). Lenvatinib versus sorafenib in first-line treatment of patients with unresectable hepatocellular carcinoma: a randomised phase 3 non-inferiority trial. Lancet 391, 1163–1173. 10.1016/S0140-6736(18)30207-1 29433850

[B19] Kulik L. El-Serag H. B. (2019). Epidemiology and management of hepatocellular carcinoma. Gastroenterology 156, 477–491. 10.1053/j.gastro.2018.08.065 30367835 PMC6340716

[B20] Langfelder P. Horvath S. (2008). WGCNA: an R package for weighted correlation network analysis. BMC Bioinformatics 9, 559. 10.1186/1471-2105-9-559 19114008 PMC2631488

[B21] Li T. Fan J. Wang B. Traugh N. Chen Q. Liu J. S. (2017). TIMER: a web server for comprehensive analysis of tumor-infiltrating immune cells. Cancer Res. 77, e108–e110. 10.1158/0008-5472.CAN-17-0307 29092952 PMC6042652

[B22] Lin G. W. Xu C. Chen K. Huang H. Q. Chen J. Song B. (2020). Genetic risk of extranodal natural killer T-cell lymphoma: a genome-wide association study in multiple populations. Lancet Oncol. 21, 306–316. 10.1016/S1470-2045(19)30799-5 31879220

[B23] Linch S. N. McNamara M. J. Redmond W. L. (2015). OX40 agonists and combination immunotherapy: putting the pedal to the metal. Front. Oncol. 5, 34. 10.3389/fonc.2015.00034 25763356 PMC4329814

[B24] Macek Jilkova Z. Aspord C. Decaens T. (2019). Predictive factors for response to PD-1/PD-L1 checkpoint inhibition in the field of hepatocellular carcinoma: current status and challenges. Cancers 11, 1554. 10.3390/cancers11101554 PMC6826488 31615069

[B25] Mahmoud S. M. Paish E. C. Powe D. G. Macmillan R. D. Grainge M. J. Lee A. H. (2011). Tumor-infiltrating CD8+ lymphocytes predict clinical outcome in breast cancer. J. Clin. Oncol. 29, 1549–1555. 10.1200/JCO.2010.30.5037 21483002

[B26] Newman A. M. Liu C. L. Green M. R. Gentles A. J. Feng W. Xu Y. (2015). Robust enumeration of cell subsets from tissue expression profiles. Nat. Methods 12, 453–457. 10.1038/nmeth.3337 25822800 PMC4739640

[B27] Nicholson S. E. Oates A. C. Harpur A. G. Ziemiecki A. Wilks A. F. Layton J. E. (1994). Tyrosine kinase JAK1 is associated with the granulocyte-colony-stimulating factor receptor and both become tyrosine-phosphorylated after receptor activation. Proc. Natl. Acad. Sci. U.S.A. 91, 2985–2988. 10.1073/pnas.91.8.2985 7512720 PMC43499

[B28] Orhan A. Vogelsang R. P. Andersen M. B. Madsen M. T. Hölmich E. R. Raskov H. (2020). The prognostic value of tumour-infiltrating lymphocytes in pancreatic cancer: a systematic review and meta-analysis. Eur. J. Cancer 132, 71–84. 10.1016/j.ejca.2020.03.013 32334338

[B29] Qin S. Ren Z. Meng Z. Chen Z. Chai X. Xiong J. (2020). Camrelizumab in patients with previously treated advanced hepatocellular carcinoma: a multicentre, open-label, parallel-group, randomised, phase 2 trial. Lancet Oncol. 21, 571–580. 10.1016/S1470-2045(20)30011-5 32112738

[B30] Rearden A. (1994). A new LIM protein containing an autoepitope homologous to “senescent cell antigen. Biochem. Biophys. Res. Commun. 201, 1124–1131. 10.1006/bbrc.1994.1822 7517666

[B31] Riaz N. Havel J. J. Makarov V. Desrichard A. Urba W. J. Sims J. S. (2017). Tumor and microenvironment evolution during immunotherapy with Nivolumab. Cell 171, 934–949. 10.1016/j.cell.2017.09.028 29033130 PMC5685550

[B32] Sekino Y. Han X. Babasaki T. Goto K. Inoue S. Hayashi T. (2020). Microtubule-associated protein tau (MAPT) promotes bicalutamide resistance and is associated with survival in prostate cancer. Urol. Oncol. S1078-1439, 30191–30195. 10.1016/j.urolonc.2020.04.032 32430253

[B33] Takahashi S. (2011). Downstream molecular pathways of FLT3 in the pathogenesis of acute myeloid leukemia: biology and therapeutic implications. J. Hematol. Oncol. 4, 13. 10.1186/1756-8722-4-13 21453545 PMC3076284

[B34] Tibshirani R. (1997). The lasso method for variable selection in the Cox model. Stat. Med. 16, 385–395. 10.1002/(sici)1097-0258(19970228)16:4<385::aid-sim380>3.0.co;2-3 9044528

[B35] Tong Y. Merino D. Nimmervoll B. Gupta K. Wang Y. D. Finkelstein D. Cross-species genomics identifies TAF12, NFYC, and RAD54L as choroid plexus carcinoma oncogenes. (2015) 27: 712–727. 10.1016/j.ccell.2015.04.005 PMC4458854 25965574

[B36] Xu X. Tan Y. Qian Y. Xue W. Wang Y. Du J. (2019). Clinicopathologic and prognostic significance of tumor-infiltrating CD8+ T cells in patients with hepatocellular carcinoma: a meta-analysis. Medicine (Baltimore) 98, e13923. 10.1097/MD.0000000000013923 30633166 PMC6336640

[B37] Zhu A. X. Finn R. S. Edeline J. Cattan S. Ogasawara S. Palmer D. (2018). Pembrolizumab in patients with advanced hepatocellular carcinoma previously treated with sorafenib (KEYNOTE-224): a non-randomised, open-label phase 2 trial. Lancet Oncol. 19, 940–952. 10.1016/S1470-2045(18)30351-6 29875066

[B38] Zhu Z. Li L. Xu J. Ye W. Chen B. Zeng J. (2020). Comprehensive analysis reveals a metabolic ten-gene signature in hepatocellular carcinoma. PeerJ 8, e9201. 10.7717/peerj.9201 32518728 PMC7258935

